# The Whys and Hows of Aesthetic Medicine: Anamnesis, Skin Assessment and Instrumental Evaluation by A Specific, Update Protocol (Mediskin Check‐Up)

**DOI:** 10.1111/jocd.70480

**Published:** 2025-10-15

**Authors:** E. Bartoletti, M. Secchi, E. Fulgione, N. Fraone, M. Veraldi, D. Feleppa, L. Cavalieri

**Affiliations:** ^1^ Ambulatory Service of Aesthetic Medicine for Psychophysical Well‐Being in Pathology Isola Tiberina Hospital—Gemelli Isola Rome Italy; ^2^ International School Carlo Alberto Bartoletti Foundation, Università Cattolica del Sacro Rome Italy; ^3^ Dermatology Clinic Luigi Vanvitelli University of Campania Naples Italy; ^4^ International School Carlo Alberto Bartoletti Foundation Rome Italy; ^5^ Private Practice Rome Italy; ^6^ Department of Plastic and Reconstructive Surgery Azienda Ospedaliera San Camillo—Forlanini Rome Italy

**Keywords:** aesthetic medicine, cosmetic schedule, instrumental skin evaluation, Mediskin check‐up, skin biotype, standardization

## Abstract

**Background:**

Skin evaluation in Aesthetic Medicine requires standardized methods to ensure reproducibility. The Mediskin check‐up protocol, first introduced in the late 1970s, provided a systematic approach to skin assessment in non‐pathological contexts. Advances in medicine and cosmetology have since required an update of this methodology.

**Aims:**

To present the updated Mediskin check‐up protocol and demonstrate its value for accurate diagnosis and personalized treatment planning in Aesthetic Medicine.

**Patients/Methods:**

The revised protocol integrates updated anamnesis, clinical examination, and instrumental evaluation under standardized conditions to ensure reproducibility.

**Results:**

The protocol allows for the identification of skin biotypes (normal, seborrheic, dry, or sensitive), defined by combined anamnesis, clinical findings, and instrumental parameters. It establishes a reproducible diagnostic framework that supports follow‐up assessments and individualized treatment regimens.

**Conclusions:**

The updated Mediskin protocol provides a standardized and reproducible method for skin evaluation in Aesthetic Medicine. By integrating clinical and instrumental data, it enhances diagnostic accuracy, guides personalized therapy, and supports safe and effective patient care.

## 1 Introduction

The skin is a highly structured organ that plays a vital role in protecting and serving the entire body system [1]. Some areas, such as the face, are cosmetically sensitive regions where certain processes are most clearly manifested (e.g., aging) [2]. The anatomical and functional integrity of the component, as well as the overall skin system is crucial. Eventual impairments are reflected in clinical signs and symptoms and/or alterations in instrumental analysis [2, 3].

Hence, in the medical field, there is a need for reproducible and standardized methods that collect clinical and instrumental evaluations, family medical histories, and patients' lifestyles. Adopting a methodological protocol in Aesthetic Medicine is fundamental for precise diagnosis and treatment [4]. This working method was developed at the end of the 1970s by Dr. Carlo Alberto Bartoletti, an aesthetic physician, and Dr. Gaston Ramette, a cosmetologist. For the first time, they established a precise protocol for the evaluation of skin without dermatological pathologies: the Mediskin check‐up [5].

Years of innovation in medicine have demanded an update in the protocol.

Here we present an update in the methodology, providing a detailed description and rationale for each step.

## 2 Materials and Methods

The revision of the Mediskin check‐up protocol has been approved by the Scientific Committee of the Italian Society of Aesthetic Medicine (SIME), a scientific society accredited by the Italian Ministry of Health.

### 2.1 Instrumental Evaluation

Instrumental evaluation of skin parameters was performed using a validated multifunctional diagnostic device (Derma Unit SSC 3, Courage & Khazaka, Cologne, Germany), which integrates a Corneometer, Sebumeter, and Skin‐pH‐Meter [6]. Measurements included hydration, sebum content, and pH, recorded at standardized anatomical sites on the face and body.

The Corneometer assessed skin hydration by measuring changes in the dielectric constant of the stratum corneum and enables detection of even minimal moisture variations via a precision capacitance sensor. It operates with a fast measurement (~1 s), ensuring minimal occlusion and reliable readings from the outer 10–20 μm of the stratum corneum, while constant spring‐loaded pressure enhances reproducibility.

The Sebumeter quantified sebum based on grease‐spot photometry. An opaque mat tape placed into contact with the skin for 30 s becomes transparent in proportion to the amount of surface lipids. The resulting transparency is measured by a photocell, the light transmission correlating with sebum content, and is displayed in Sebumeter units (approximately μg/cm^2^). The tape is oil‐specific and unaffected by water, and a spring mechanism ensures consistent measurement pressure.

The Skin‐pH‐Meter employs a high‐quality combined glass electrode system, housing both an H^+^ ion‐sensitive electrode and a reference electrode within one glass body, fully integrated with measurement electronics via a probe handle (following standard design by Courage and Khazaka).

### 2.2 Lactic Acid Sting Test (LAST)

The lactic acid sting test was performed by applying a 15% aqueous lactic acid solution to one cheek using a cotton pad, while sterile water was simultaneously applied to the contralateral side as a placebo. The test was conducted in a blinded manner for the subjects. Sensations were evaluated at 1, 3, and 5 min after application. Participants reported the intensity of discomfort, such as stinging, tingling, itching, tightness, burning, or pain, using a 4‐point scale (0 = absent, 1 = mild, 2 = moderate, 3 = severe).

### 2.3 Dermographism Assessment

Dermographism, also known as dermatographism or “skin writing,” was assessed by applying controlled mechanical stimulation to the skin, by sliding a blunt tip across the skin of the décolleté to form an X. This condition is characterized by transient wheals and erythema following rubbing, pressure, or scratching. The reaction reflects an abnormal activation of cutaneous mast cells, which release histamine and other pro‐inflammatory mediators, leading to vasodilation, edema, and visible wheal‐and‐flare responses.

### 2.4 Wood's Lamp Examination

A Wood's lamp examination was performed using ultraviolet light to evaluate skin conditions, including the presence and depth of hyperpigmentation, the degree of comedone oxidation, scaling, and areas of vitiligo.

## 3 Results

### 3.1 Protocol Aim

The objective of the updated Mediskin check‐up protocol aims to formulate a diagnosis on the “skin biotype”. This diagnostic element is essential for planning an Aesthetic Medicine therapeutic regimen and to have a baseline for follow‐up evaluations [4, 5].

The “skin biotype” is the result of the Mediskin check‐up protocol and is not just a one‐way output. Rather, it is a complex, individualized, detailed report obtained from subjects' personal and family medical histories, daily habits, and lifestyles (with a potential impact on the skin), as well as clinical assessments and instrumental analyses [4, 6].

### 3.2 Standardization and Subject Preparation

Factors such as the cleaning procedure, the regional variation in temperature and humidity, can impact the measurement of skin parameters [7].

Regarding skin moisture measurement, environmental temperature and humidity are important factors.

Another important factor is the application of a standardized cleaning procedure for the skin. Even after mild cleaning, it is necessary to wait at least 5 h before capacitance measurement can be carried out [7].

The choice of the measurement site is another key point, as regional variations may be due to differences in the thickness of the stratum corneum and the number and activity of sweat glands [7].

Lastly, age appears to be an important characteristic influencing skin hydration, whereas gender has no effect [7].

For the reasons just described, some simple instructions should be given to subjects who plan an Aesthetic Medicine visit to have the most reproducible conditions of assessment:

- No direct sun exposure to the face and neck area in days preceding the visit, and no tanning bed use within the previous 15 days.
- Cleanse the face and the body with the usual dermocosmetic formula the night before and avoid lotions.
- No application of topical treatments and/or make‐up and no cleansing the morning of the scheduled visit [6].

The environment of the doctor's room should be controlled, with a temperature of 21°C (±1°C) and a humidity of 50% (±10%) [8]. The subject requires an acclimatization period of 15–30 min [6].

These recommendations are essential before the skin check‐up, as the measurements aim to assess the skin's ability to restore the hydrolipidic film after its removal with the cleansing solution usually used by the patient.

### 3.3 Anamnesis

The questions follow the pattern of the medical anamnesis: family medical history; personal physiological history, including information on lifestyle (diet, physical activity, sleep, alcohol, smoke, and sun exposure), gynecological data when applicable (menstrual cycle, pregnancies, and menopause); personal remote and recent pathological medical history, including possible allergies and/or intolerances; personal cosmetic habits and routines (cleansing, topical formulas, and sun protection) and clinical history of cutaneous reactions of any type; skin and adnexa self‐evaluation (cutaneous sensations and subjective conditions experienced) [6].

### 3.4 Clinical Exam

First, a general evaluation is performed (including height, weight, muscle status, and lympho‐venous circulation functionality). Then, a local examination is conducted (including inspection and palpation of skin, scalp, and nails) followed by a clinical assessment of skin quality and phototype classification according to Fitzpatrick's scale [6].

Assessment of the degree of photodamage is also an integral part of the patient's clinical evaluation, given the well‐established role of sun exposure in accelerating skin aging and skin damage.

Clinical signs of photodamaged skin include markedly increased skin roughness, mottled hyperpigmentation, loss of elasticity, wrinkling, and the development of solar lentigines and actinic keratosis [8, 9, 10].

The evaluation of photodamage is normally conducted using the Rubin Classification, which is based on the histologic depth of visible clinical changes.

Rubin classification:

- Level 1: clinical signs are due to alterations in the epidermis only. Most abnormalities involve skin pigmentation and texture, including freckles, lentigines, and a dull rough skin texture due to the increased thickness of the stratum corneum.
- Level 2: clinical signs are due to alterations in the epidermis and papillary dermis and are also often related to abnormal pigmentation. Textural and pigmentary changes are more marked than in level 1. These patients may have actinic keratosis, liver spots (senile lentigines or flat seborrheic keratosis), and a definite increase in wrinkling. The increased wrinkling is usually seen in the infraorbital areas and lateral to the nasolabial groove, where the skin may appear atrophic and crinkled.
- Level 3: clinical signs are due to alterations in the epidermis, papillary dermis, and reticular dermis. The most severe form of photodamage, level 3, is associated with many of the clinical changes seen in levels 1 and 2. These patients, however, also have marked wrinkling, which is usually associated with a thickened leathery appearance and texture, as well as a yellowish tint of the skin. In addition, the skin of some patients has a pebbly texture and scattered open comedones [11].

### 3.5 Instrumental Evaluation

The physiological balance of the skin is also expressed through the state of well‐being of the hydrolipid film, which can be evaluated through skin measurements such as the degree of sebometry, hydration, and pH. Table 1 summarizes the measurements and skin tests performed during the initial exam.

**TABLE 1:**
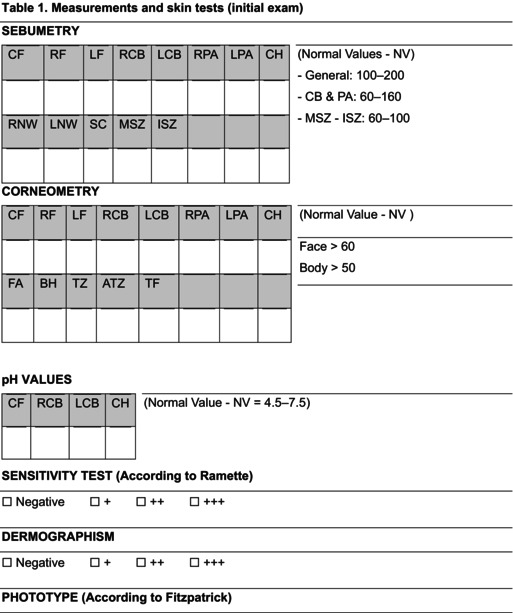
Measurements and skin tests (initial exam).

Abbreviations: ATZ, anterior tibial zone; BH, back of hand; CF, center of forehead; CH, chin; FA, forearm; ISZ, interscapular zone; LCB, left cheekbone; LF, left forehead; LNW, left nasal wing; LPA, left preauricular zone; MSZ, mid‐sternal zone; RCB, right cheekbone; RF, right forehead; RNW, right nasal wing; RPA, right preauricular zone; SC, scalp; TF, top of foot; TZ, trochanteric zone.

Measurement of multiple skin parameters is performed with a validated diagnostic medical device (Derma Unit SCC3 apparatus, Courage & Khazaka, Cologne, Germany) [6, 12].

The Derma Unit SSC3 is a classic compact device with a digital display for Sebumeter/Corneometer/Skin‐pH‐Meter in one housing, and it is suitable for characterizing the hydrolipidic film of the skin.

Several hundreds of studies have been performed on the Corneometer, the Sebumeter, and the Skin‐pH‐Meter, which are worldwide established as standard equipment [12].

The measuring principle [12]:

- The Corneometer can determine the change of the dielectric constant caused by moisture on the skin surface.
- The Sebumeter is a grease spot photometer. The foil of the Sebumeter cassette is brought into contact with skin or scalp for 30 s at an automatically standardized pressure. The foil becomes more transparent as the sebum content of the measured area increases. The detected transparency is measured by a photodiode.
- The Skin‐pH‐Meter is based on a high‐quality combined electrode, in which both H^+^ ion‐sensitive electrodes and the additional reference electrode are placed in one glass housing. This is connected to a probe handle that contains the measurement electronics.

Measurements within the normal range [13]:

- Sebometry (μg/cm^2^)
  ○ forehead, t‐zone, scalp: 100–200
  ○ Scalp: 40–100
  ○ Cheek, eyelid, nape: 70–180
  ○ Corner of mouth, upper parts of body, back, neck: 55–130
  ○ Limbs, hands, elbows: > 6
- Hydration (in arbitrary units between 0 and 130):
  ○ sufficiently hydrated > 60 face; > 50 body
- pH:
  ○ Women: 4.5; 5.0; 5.3; 5.5
  ○ Men: 4.3; 4.5; 5.0; 5.3; 5.5

The measurements are taken, respectively, as follows [6]:

- Face:
  ○ Skin moisture at the frontal region (3 points: 1 centrally and 2 laterally right and left at the correspondence of the temporal ridges), on the zygomatic prominences (2 points, 1 per side at the Hinderer lines intersection), on the chin (1 at the cutaneous projection of the mental tubercle), and at the preauricular region medially to the tragus (2 points, one on each ear).
  ○ Sebum is evaluated at the same points, plus the ala of the nose (2 points, 1 per side) and on the scalp (2 cm centrally internally to the hairline).
- Body:
  ○ Skin moisture on the volar side of the right forearm, on the central area of the dorsal side of the right hand, in the area corresponding to the cutaneous projection of the trochanter area, at the anterior of the tibial region, and the dorsal area of the right foot.
  ○ Sebum is evaluated at the midsternal and interscapular regions.

In addition to these parameters, dermographism and lactic acid sting test (LAST) are performed [6, 14]. The examination is then completed with observation under cold light and a Wood's lamp to determine the presence and depth of pigmentation in skin hyperpigmentation, to observe the degree of comedone oxidation, and to assess the presence of scales and areas of vitiligo [6, 15].

### 3.6 Conclusions

Once all the data are collected, the doctor draws a conclusion on the current skin state of both body and face areas. The conclusion describes the main cutaneous features and highlights relevant aesthetic issues, including dry and seborrheic skin. According to the skin status (“biotype”) and the subjects' needs, an Aesthetic Medicine Program will follow [4]. In case cutaneous lesions are found, a dermatological visit is recommended for the subject [6].

The following criteria [6, 15] can be used to describe the biotypes:

- Normal skin: a skin “in balance”, where the clinical evaluation does not detect features to note, and the instrumental measurements are within the normal range in all the measured area.
- Skin with an increased content of superficial lipids (up to seborrhea): Seborrheic skin is characterized by increased sebum production by sebaceous glands.
  Particularly on the “T‐zone” the skin is oily, greasy, and the follicular ostia are dilated. Open or closed comedones can be detected [6].
  Seborrhea is a condition that promotes the development of acne lesions associated with the increase in sebum of very irritating free fatty acids from the breakdown of triglycerides by bacterial lipases [16].
  Finally, seborrhea is linked to the development of another dermopathy, seborrheic dermatitis, which is characterized by the development of erythematous‐desquamative lesions in typical sites such as the face, scalp, and trunk. In the determinism of the disease, the role of *Pityrosporum ovale*, a saprophytic yeast, is certainly important, but also a quantitative and qualitative change in surface lipids that results in an inability of the skin to retain water [16].
  Sebometry usually registers higher rates, although lower values can be found too, especially if the subject has used anti‐lipid enhancing formulas, contraceptive pills, or if menopause occurs. Corneometry could be in range or decreased, as when subjects do not hydrate the skin in the morning fearing the worsening of the appearance of oily skin. Cutaneous pH values may be normal or alkaline due to imbalances in bacterial microflora [6].
- Dry skin: Dry skin is the clinical manifestation of skin that is deficient in water and/or lipids. The skin could be dull and rough with scaling; the follicular ostia are restricted and barely visible. It should be noted that current literature defines dry skin almost exclusively in terms of dehydration, rarely addressing cases characterized by a deficiency in surface lipids.
  It is possible to distinguish three different types of dry skin [6, 16]:
  ○ Pathological forms due to genetic causes (ichthyosis), inflammatory causes (eczema), keratinization abnormalities (psoriasis), and systemic diseases.
  ○ Non‐pathological constitutional forms (xerosis vulgaris and fragile skin).
  ○ Acquired forms resulting from the exposure to environmental conditions (wind, environments with improper air conditioning), use of medications (retinoids, estroprogestinics, diuretics, cholesterol‐lowering drugs), prolonged exposure to chemicals, UV‐induced skin aging, and altered dietary conditions.
  Larger or only localized areas can be included in this biotype. According to the underlying mechanism of cutaneous dryness, sebometry and corneometry can have different values. A lack of hydration could be associated with higher water loss and normal sebum assessment, as well as unstable pH, whereas a reduced sebum secretion is related to standard corneometry and lower pH values [6].
- Sensitive skin: sensitive skin is defined as a syndrome characterized by unpleasant sensations, such as stinging, burning, pain, pruritus, or tingling, in response to stimuli that should not normally provoke such reactions [17]. These symptoms occur without visible lesions attributable to any skin disease, and the skin may appear normal or present with erythema. Although originally described as a facial condition, it is now clear that sensitive skin is not restricted to the face; it may also occur on any area of the body, scalp, and genital area [17].
  Sensitive skin is recognized as an independent syndrome, though it may coexist with dermatological conditions such as atopic dermatitis (AD) or rosacea, or with an atopic predisposition [17]. It is not confined to individuals with dry skin, yet the two frequently overlap, sharing similar triggers and often presenting in combination [14].
  Despite growing recognition, the relationship between sensitive skin and barrier function remains incompletely understood. Proposed mechanisms include increased barrier permeability, xerosis, reduced natural moisturizing factor, and elevated transepidermal water loss, with barrier dysfunction in diseases such as AD and rosacea possibly contributing further [18]. Beyond barrier alterations, neurogenic and immune‐related mechanisms are also implicated [19]. Together, these observations support the view that sensitive skin is multifactorial, shaped by a complex interplay of genetic predisposition and environmental exposures.

### 3.7 Aesthetic Medicine Program

Once the subject biotype has been determined, an Aesthetic Medicine therapeutic regimen is recommended: the management will include a well‐defined and personalized cosmetic schedule where cleansing, photoprotection, and dermocosmetic formulas are indicated [6]. If necessary, a program of ambulatory treatments such as peelings, fillers, or other aesthetic interventions will be implemented [6]. The subject will receive indications for laboratory exams or for other specialized medical visits in case further information or additional medical referrals are required for comprehensive care [4, 5, 6].

## 4 Discussion

The skin is a complex structure with multiple, essential functions, serving and protecting our body [1]. It safeguards individual wellness and health by protecting and regulating body contents, fluids, and temperature, supporting injury repair, immune processes, and vitamin D metabolism [1, 20]. Facial skin has an impact on subjective well‐being and quality of life, and its role in the psychological sphere is undeniable [21].

Anatomically, the epidermis represents the epithelial, external component with specifically distributed cellular layers, which have a bottom‐up differentiation and a crucial role in the barrier function, supported by the so‐called brick‐and‐mortar structure [1, 22]. Keratinocytes are not the only elements that contribute to skin functions: melanocytes play a role in endogenous UV protection, sebaceous and sweat glands secrete lipid‐rich or water‐rich content important for cutaneous homeostasis, blood vessels in the dermis contribute to temperature regulation and nutrient supply, nervous fibers represent the starting point for sensation transmission to the central nervous system, and the microbiome takes part in the skin system with numerous biological effects [1].

Clinical and instrumental evaluations allow for the assessment of different characteristics and integrity of the skin, a topic that has already been addressed in the scientific community from both a pathological and aesthetic point of view [10].

Certainly, nowadays many health care professionals are approached by patients as aesthetic advisors, and sometimes the conditions are part of the dermatological or plastic surgery area of expertise and competence, where a specialized MD is required to diagnose and treat the disease. In other cases, a specific expertise in aesthetic medicine may be the appropriate referral, as this medical area has grown and progressed over time to become a specialized medical discipline, with a parallel demand for highly trained personnel [23]. Aesthetic Medicine finds its roots in the Medical Art itself, where solid, scientific bases are key drivers for diagnosis and treatment [4, 6]. The interaction of Aesthetic Doctors with their patients represents an important step in their journey, and the global examination, including anamnesis, skin, and instrumental evaluation, allows patients not only to access an accurate aesthetic diagnosis with an appropriate therapeutic regimen but also counseling on a healthy lifestyle and, when necessary, a referral to other specialized doctors for emerging health issues [4]. Cosmetologists have already been identified as potential stakeholders to promote pro‐health activities [24], and aesthetic doctors share this responsibility. Therefore, the aim of Aesthetic Medicine extends to providing patients with comprehensive, holistic care. To achieve this goal, a rigorous methodology is required, with all key clinical elements systematically collected according to a defined protocol [4, 5, 6]. This work has been carried out since the end of the 1970s by Doctors Carlo Alberto Bartoletti and Gaston Ramette, and this method continues to be valid and modern, supported by innovations in medicine and cosmetology [4, 5]. The skin check‐up was included in 1990 among the morphofunctional assessments that are part of the more comprehensive aesthetic medicine check‐up, which also includes psychological evaluation, morpho‐anthropometric assessment, angiological evaluation of the lower limbs, ultrasound evaluation of adipose tissue, postural assessment, and hematochemical evaluation [6].

Regarding the differences between this updated check‐up protocol and the previous version, the earlier protocol relied on Cesarini's classification system for determining skin type, whereas the updated version now uses the Fitzpatrick classification. Another key addition is the assessment of photoaging using Rubin's classification. Additionally, Mediskin's parameters of normality are now based on the Derma Unit SSC 3, replacing the older 825 model.

The current version of the Mediskin check‐up protocol aims to be a medical, scientific *modus operandi* where each single question asked and parameter registered is not simply an output, but a clue element with its clinical significance and implications in both diagnostic and therapeutic aspects. The delivery of a complete check‐up must be preceded by specific training including skin and adnexa anatomy and physiology, instrumental knowledge, cosmetology, and cosmetics education. Since 1990, an International School has provided a four‐year, post‐graduate training course on all the main topics of Aesthetic Medicine [25]. The *alumni* deliver the protocol in their daily practice as Aesthetic Doctors in both baseline and follow‐up visits [25]. The results of their clinical practice can provide an overview of the protocol results (e.g., according to subjects' age), and the authors aim to revisit this topic to address the limitations of the current publication, as no dataset has yet been provided.

To conclude, Aesthetic Medicine “whys” and “hows” are not simple open questions in the scientific panorama. The aims (“whys”) cover a multitude of community needs, supporting a scientifically personalized approach also for aesthetic issues, counseling, and referral. On the other hand, the methodology (“hows”) can benefit from a standardized protocol that represents the fundamental basis for precise diagnosis and treatment.

## Author Contributions

Conceptualization: E.B., M.S., E.F. Methodology: E.B., M.S., E.F. Writing review, editing and approval to submit: All Authors.

## Consent

Informed written consent has been given by all the subjects included in the protocol.

## Conflicts of Interest

The authors declare no conflicts of interest.

## Data Availability

The data that support the findings of this study are available from the corresponding author upon reasonable request.
